# Administering immunotherapy after anti-vascular targeted therapy improves overall survival of patients with metastatic clear cell renal cell carcinoma

**DOI:** 10.7150/jca.96514

**Published:** 2024-06-17

**Authors:** Zhiwei Hou, Long Lai, HuaGuo Wu, Benkui Zou, Ni Xu, Dongyuan Zhu, Xiaokun Wang, Hui Zhang

**Affiliations:** Affiliated Cancer Hospital of Shandong First Medical University, Shandong Cancer Hospital and Institute, Shandong First Medical University and Shandong Academy of Medical Science, Jinan, Shandong, China.

**Keywords:** metastatic clear cell renal cell carcinoma, tyrosine kinase inhibitor, immune checkpoint inhibitors, PD-1

## Abstract

Background: The Food and Drug Administration of the United States has approved several drugs for treating advanced metastatic renal cell carcinoma, including anti-vascular tyrosine kinase inhibitors (TKIs) and immune checkpoint inhibitors (ICIs). Options for first-line therapy include monotherapy or combination therapy. However, selecting a suitable first-line and second-line treatments to improve overall survival remains an unresolved issue.

Objective: To evaluate the overall survival (OS) and progression-free survival (PFS) of patients with metastatic clear cell renal cell carcinoma (mRCC). Patients were divided into several grouped according to the treatment sequence of TKI and anti PD-1 administration. The overall survival benefit was evaluated based on the order of administration of anti PD-1 and TKI.

Patients and Methods: In this retrospective propensity-matched cohort study, we identified 135 patients with mRCC treated at the Affiliated Cancer Hospital of Shandong First Medical University from January 1, 2017, to December 31, 2022. These patients had received anti PD-1 treatment as part of their first or second line of therapy. Statistical analysis was performed from June 1, 2023, to August 1, 2023. The primary outcome measure was OS, from the date of diagnosis to death or the last follow-up. PFS was monitored during treatment. Survival analyses were conducted using Cox proportional hazards regression and Kaplan-Meier estimates. By comparing the complete treatment course of patients, the survival of patients in different groups was compared according to the number of immunotherapy lines.

Results: The final cohort comprised 135 patients, of whom 84 received first-line therapy with anti PD-1 (include 6 patients treated with anti PD-1 (tislelizumab, carrelizumab, toripalimab or sintilizumab) alone and 78 patients treated with anti PD-1 combined with anti-vascular TKI (axitinib, sunitinib, solfanitinib or pazopanib)). The remaining 51 patients were treated with anti PD-1 as second-line therapy following an initial regime of TKIs. Patients were initially categorized based on whether anti PD-1 were used in the first-line treatment. It was observed that the OS of patients receiving first-line targeted therapy was higher than those receiving first-line immunotherapy, with a median OS of 33 months versus 15 months. To investigate this outcome further, we refined the patient groups based on the administration sequence of anti PD-1 and TKIs in the treatment regimen. We found that the median PFS of patients with first-line treatments of TKI combined with anti PD-1 was 3.5 months, compared to 14.5 months when TKI combined with anti PD-1 followed first-line TKI (*p*=0.0092). The median PFS for second-line treatments was 6.5 months versus 15 months (*p*=0.0014). Similarly, the median OS was 16.66 months and 31.88 months, respectively (*p*=0.008).

Conclusions: This study indicates that administering immunotherapy following anti-vascular therapy significantly enhances both OS and PFS compared to other sequences of therapies. This finding provides valuable insights and robust data support for clinical decision-making regarding treatment sequencing.

## Introduction

Renal cell carcinoma (RCC) ranks the sixth most prevalent cancer among men and the eighth most common among women in the United States, contributing to 4.2% of all new cancer cases and 2.4% of cancer-related deaths annually 1. The incidence of RCC continues to rise, and the management of metastatic clear cell renal cell carcinoma (ccRCC) remains challenging, with a 5-year survival rate of only 12% 2. Anti-vascular targeted therapy has emerged as a key treatment modality for RCC, and with the advent of immunotherapy, the options for treating metastatic ccRCC have expanded. Current therapeutic approaches for metastatic ccRCC include targeted therapy, immune checkpoint inhibitors (ICIs), and combination therapies 3.

Pathological angiogenesis in RCC involves several key signaling pathways, notably the vascular endothelial growth factor (VEGF), platelet-derived growth factor (PDGF), and mammalian target of rapamycin (mTOR) pathways 4. One of the characteristics of RCC is rich in angiogenic factors, which established anti-vascular therapy as a cornerstone in its treatment. The arsenal of anti-angiogenic drugs used in RCC includes receptor tyrosine kinase inhibitors, monoclonal antibodies, and mTOR inhibitors.

The tumor microenvironment of ccRCC is distinguished by a substantial infiltration of T cells, natural killer cells, and dendritic cells 5, classifying it as an immunogenic tumor. The introduction of immune checkpoint inhibitors like pembrolizumab and nivolumab in the treatment of renal clear cell carcinoma has yielded significant therapeutic success 6, 7, leading to their gradual endorsement as recommended immunotherapy options. Although the efficacy of immunotherapy in treating metastatic ccRCC has been established, optimal sequencing for administration of these drugs to maximize patient survival benefits remains unspecified in prior studies. Therefore, there is a critical need to strategically evaluate various treatment modalities to enhance patient survival outcomes.

## Materials and Methods

### Patient characteristics and clinical data

We conducted a retrospective analysis in 135 patients with metastatic clear-cell renal-cell carcinoma who had previously received immunotherapy between January 1, 2016, and December 31, 2022. The last follow-up date was May 31, 2023, by which time 57 patients had passed away. The patients included in our study had histologically confirmed metastatic clear-cell renal-cell carcinoma that was determined on the basis of TNM staging and had received immunotherapy. Exclusion criteria were patients with other cancers, coexisting infections or autoimmune diseases.

Among these patients, 84 received first-line treatment with anti PD-1 (include 6 patients were treated with anti PD-1 alone and 78 patients were treated with combination of anti PD-1 and TKI), including tislelizumab, carrelizumab, toripalimab or sintilizumab. The remaining 51 patients were treated with anti PD-1 or anti PD plus TKI as second-line therapy following progression with anti-vascular tyrosine kinase inhibitors (TKIs) therapy, comprising axitinib, sunitinib, solfanitinib or pazopanib.

Patient characteristics were summarized using frequency (%) for categorical variables and median (range) for continuous variables. Baseline characteristics, including patient and tumor details, were extracted from the dataset. Continuous variables were compared using analysis of variance testing, while the χ^2^ test of independence and the Kruskal-Wallis test were employed for normally and nonnormally distributed categorical variables, respectively. Variables considered in the formulation of propensity scores included age, sex, IMDC (International Metastatic Renal Cell Carcinoma Database Consortium) score, and cT stage.

The survival analysis was pre-designed to incorporate variables from the postmatching univariable analysis into the univariate logistic regression for OS, stratified by treatment groups. Statistical analyses were performed using IBM SPSS Statistics 25 and GraphPad Prism 9.

## Results

Patients and disease characteristics are detailed in Table 1. Our study involved 135 patients, with 84 patients receiving first-line anti PD-1 (include 6 patients treated with anti PD-1 alone and 78 patients were combined with TKI). Among these, 40 patients were aged ≥60 years and 44 were <60 years old. The gender distribution included 62 males and 22 females. IMDC classification was based on the time from diagnosis to initial treatment, hemoglobin count, absolute neutrophil count, platelet count, blood calcium concentration, and Karnofsky performance status, categorized 22 patients in the low-risk group, 55 in the medium-risk group, and 7 in the high-risk group. The distribution of cT stages was as follows: T1 in 25 cases, T2 in 26 cases, T3 in 15 cases, T4 in 5 cases, and Tx in 13 cases. In the cohort of 51 patients receiving second-line immunotherapy, 29 were aged ≥60 years and 22 were <60 years old, with a gender distribution of 44 males and 7 females. The risk stratification for this group comprised 13 low-risk, 32 medium-risk, and 6 high-risk cases. The cT stage distribution was T1 in 14 cases, T2 in 15 cases, T3 in 11 cases, T4 in 4 cases, and Tx in 7 cases. We conducted a differential analysis on the differences in baseline characteristics of patients and did not find any statistically significant factors that affected patient survival (Table 1). Data were segregated based on the use of first-line immunotherapy. Univariate logistic regression analysis revealed a statistically significant difference between first-line and second-line immunotherapy groups (*p*=0.002, < 0.05) (Table 2).

Adverse events during first-line and second-line treatment were recorded in the two groups. The main adverse events included bone marrow suppression, elevated liver enzymes, hypertension, proteinuria, deep venous thrombosis, feeble and hand-foot syndrome. In the first-line immunotherapy group, in first-line treatment bone marrow suppression and elevated liver enzymes, hand-foot syndrome are more severe than in the second-line group; in the second-line immunotherapy group, in first-line treatment bone marrow suppression and deep venous thrombosis are more severe than in the second-line group (Supplementary Table 1). We focused on the incidence of adverse events associated with immunotherapy combined with targeted therapy in the first-line treatment or second-line treatment. We found that there was no significant difference in the incidence of adverse events between the two groups, whether in first-line or second-line combined therapy (Table 3).

**Figure 1:**
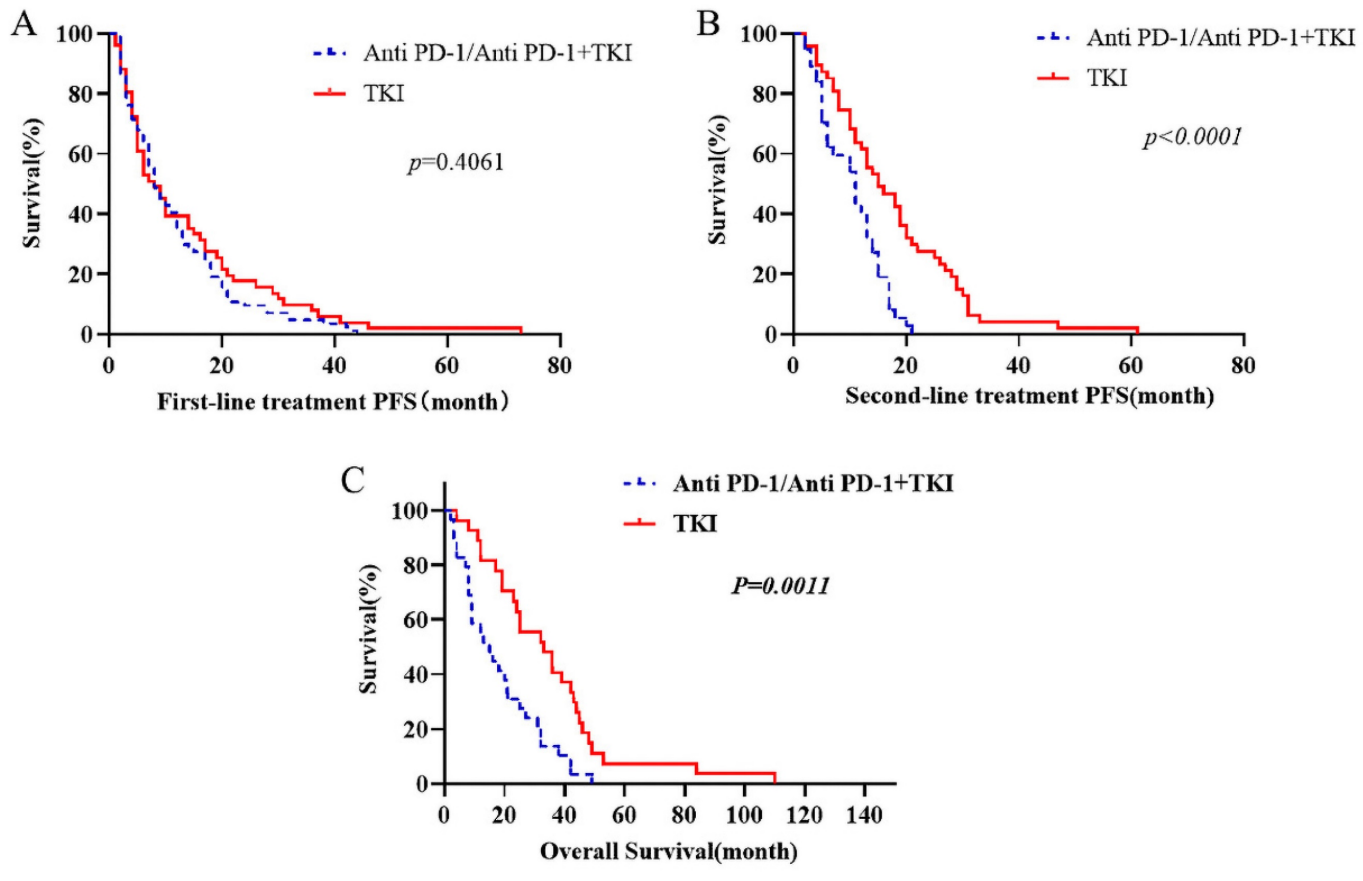
The OS of first-line targeted therapy was compared. First-line immunotherapy includes treated with anti PD-1 alone and combined with TKI. The PFS of each treatment stage was compared.

Kaplan-Meier estimates were utilized to visually represent the survival distributions. The comparative analysis indicated significant differences in OS between patients receiving first-line immunotherapy (anti PD-1 alone and combined with TKI) and those receiving first-line TKI (Fig. 1). Notably, the OS was higher in patients receiving first-line TKI compared to those with first-line immunotherapy (p=0.0011). Further analysis showed that the PFS of second-line therapy following first-line TKI was substantially greater than the PFS following progression on first-line immunotherapy (p< 0.0001). However, the difference in PFS for the first line of treatment was not significant between the two groups (p= 0.4061) (Fig. 1).

**Figure 2:**
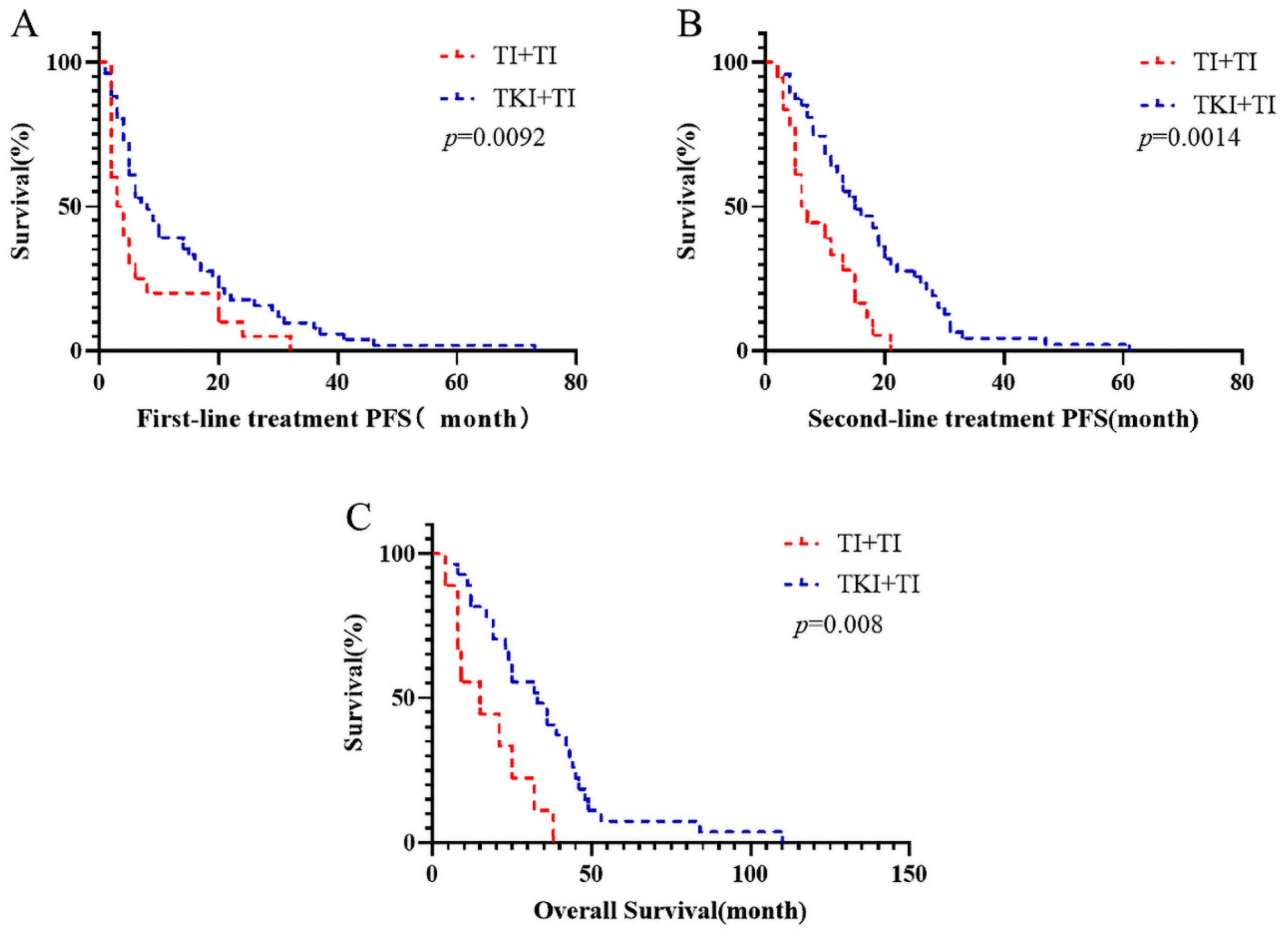
Compare the first-line immune and targeted therapy progress after a line continues to ignore united target therapy and immune targeted therapy progress after immune OS, targeted therapy of joint and PFS at each stage (TI: TKI combined Immunotherapy).

To investigate the reasons behind these outcomes, we further refined the patient grouping. Patients were categorized based on using anti PD-1 or TKIs as the first-line treatment, and the outcomes of different second-line treatment regimens were compared. It was observed that the survival of patients treated with first-line TKI in combination with anti PD-1 was not significantly different from that of patients who received first-line anti PD-1 alone (Supplementary Fig. 1). The survival outcomes of patients receiving first-line TKI were comparable to those treated with first-line anti PD-1 alone (Supplementary Fig. 2). Interestingly, we discovered that the survival of patients who began with TKI followed by a combination of anti PD-1 and TKI was superior to that of patients starting with first-line immunotherapy combined with targeted therapy. The median PFS following first-line TKI combined with anti PD-1 was 3.5 months, compared to 14.5 months when TKI combined with anti PD-1 followed first-line TKI (*p*=0.0092). The median PFS for second-line treatments was 6.5 months versus 15 months (*p*=0.0014). Similarly, the median OS figures were 16.66 months and 31.88 months, respectively (*p*=0.008) (Fig. 2).

## Discussion

The important finding of this study is that the survival of patients who began with TKI followed by a combination of anti PD-1 and TKI was superior to that of patients starting with first-line immunotherapy combined with targeted therapy. This finding provides valuable data for the management of metastatic clear-cell renal cell carcinoma. We found that initiating treatment with first-line anti-vascular targeted therapy followed by second-line immunotherapy can significantly improve patient overall survival. Additionally, administering immunotherapy subsequent to the first-line anti-vascular therapy not only enhances the effectiveness of subsequent treatment line but also improves progression-free survival. Our study provides a more effective and reasonable medication sequence for patients with metastatic clear cell renal cell carcinoma, and provides better survival for patients.

In the past few decades, our understanding of metastatic clear cell renal cell carcinoma has evolved alongside with drug development, leading to significant changes in treatment strategies. The emergence of the first generation anti-angiogenic TKIs marked a notable improvement in patient outcomes. In a study conducted by Robert J. Motzer et al., 106 patients were enrolled for second-line treatment with sunitinib, and the efficacy analysis was done in 105 patients. Among these patients, 36 of them experienced a partial response, and the median progression-free survival was 8.3 months 8. This study confirmed the efficacy of sunitinib in first-line treatment, showing it to be superior in terms of progression-free survival (the primary endpoint) compared to interferon-alpha treatment 9. Furthermore, the effectiveness of pazopanib and sorafenib in treating metastatic clear cell carcinoma has been verified in clinical trials 10, 11.

The advent of the second-generation anti-angiogenic TKIs marked another therapeutic milestone. In a study by Robert J. Motzer et al., axitinib, used as a second-line treatment for advanced renal cell carcinoma, demonstrated longer PFS compared to sorafenib 12. The study of T.K. Choueiri et al. highlighted cabozantinib, a multi-target inhibitor, as having higher safety compared to other options 13.

The introduction of immune checkpoint inhibitors (PD-1/PD-L1, CTLA-4) revolutionized the treatment of metastatic clear-cell renal cell carcinoma, offering new options for patients with progression on first-line targeted therapy, beyond just second-line targeted therapies. In a study by Robert J. Motzer et al., nivolumab (the first FDA-approved ICI agent for mRCC) was associated with superior OS and a lower rate of grade 3/4 adverse events as a second-line treatment for advanced renal cancer compared to everolimus 6. Furthermore, combination therapy with nivolumab plus ipilimumab, although less effective as a first-line treatment compared to nivolumab alone, showed promising efficacy as a second-line therapy post-nivolumab progression 14, 15.

In clinical trials, combination immunotherapy has been used as a first-line regimen. In another study by Robert J. Motzer et al., patients with untreated metastatic RCC received either pembrolizumab plus axitinib or sunitinib. The one-year OS was 89.9% in the pembrolizumab plus axitinib group versus 78.3% in the sunitinib group; PFS was 15.1 months versus 11.1 months, respectively (HR 0.69; *p*< 0.0001). The objective response rate (ORR), a secondary endpoint, was 59.3% in the experimental arm compared to 37.5% in the control group 16. Based on this study, the FDA approved pembrolizumab plus axitinib for the first-line treatment for patients with renal cell carcinoma on April 19, 2019. Combined immunotherapy has increasingly become a mainstream treatment. However, a recent study suggests that the combination of lenvatinib plus pembrolizumab versus sunitinib in patients does not seem to improve OS 17, indicating that combination immunotherapy may not always be the best first-line treatment option.

In a particular study, no significant difference in PFS was observed between targeted therapy and immunotherapy in the second-line setting among patients who had previously received first-line immunotherapy 18. This outcome suggests that early administration of immunotherapy might potentially deprive patients of more effective treatment options in subsequent lines of therapy. A recent study echoed similar findings in non-clear cell renal cell carcinoma, where the combination of immunotherapy and targeted therapy exhibited better PFS following cancer progression on first-line targeted therapy 19. These results in non-clear cell renal cell carcinoma might have implications for clear cell renal cell carcinoma, indicating potential parallels in treatment responses and strategies between these two subtypes of RCCs.

Anti-vascular therapy might enhance the efficacy of subsequent immunotherapy. Continuous treatment with anti-vascular agents induces secondary hypoxia in tissues or tumor cells, which might activate the hypoxia-inducible factor (HIF) pathway. Tumor cells adapt to this hypoxic environment by altering themselves and secreting additional pro-angiogenic factors, including EGFR, PIGF, FGF2, erythropoietin (EPO), TGF-α, IL-6, and IL-8. This adaptation can lead to resistance against anti-vascular targeted therapies 20. IL-6 and IL-8, both significant pro-angiogenic factors, are also markers of poor prognosis in mRCC and are highly expressed in patients with TKI resistance 21, 22. Hypoxia stabilizes and upregulates PD-L1 expression through HIF-2α in ccRCC 20, and HIF-1α can lead to overexpression of PD-L1 in immune cells, thereby negatively regulating cytotoxic T cells 23.

The activation of the HIF pathway by continuous anti-vascular therapy might exacerbate tissue hypoxia and promote the production of angiogenic factors, leading to resistance to anti-vascular targeted drugs and disease progression. This secondary hypoxia increases the expression of PD-1 and PD-L1, potentially enhancing the efficacy of immunotherapy. VEGF, apart from altering the tumor microenvironment (TME), might enhance the population and functionality of immunosuppressive cells such as regulatory T lymphocytes (Tregs), myeloid-derived suppressor cells (MDSCs), and M2 macrophages. It causes a loss of activity in tumor-infiltrating lymphocytes, which might promote immune evasion and tumor growth 24. VEGF can inhibit dendritic cell maturation, reduce T-cell infiltration, and promote inhibitory cells in the TME 25. It results in decreased expression of PD-1 and CTLA-4 on immune cells 26. These findings suggest that anti-angiogenic drugs can effectively enhance tumor immunogenicity, thereby improving the response to immunotherapy.

Despite the abundance of clinical data on mRCC, the absence of systematic and comprehensive medication records throughout treatment processes significantly impacts patients' OS outcomes. Our study meticulously recorded the complete treatment course of enrolled patients, enabling us to compare and analyze the most effective immunotherapy regimens.

With a deeper understanding of the pathogenesis of metastatic clear cell renal cell carcinoma, a plethora of therapeutic drugs have been developed, presenting a challenge in selecting the appropriate treatment modalities. Determining the optimal first-line treatment plan, as well as making informed decisions about subsequent treatment strategies for progressive diseases, requires careful consideration. Clinicians face the critical task of selecting a reasonable and effective treatment plan from various available options, and the finding of this study would help clinical decision-making.

## Conclusions

In this retrospective study, we discovered that administering immunotherapy following anti-vascular targeted therapy significantly enhances patient overall survival. Additionally, implementing immunotherapy after first-line anti-vascular therapy not only improves the effectiveness of subsequent lines of treatment but also positively impacts the PFS of patients. This finding highlights the strategic importance of sequencing therapy in metastatic clear cell renal cell carcinoma, emphasizing the potential benefits of integrating immunotherapy following anti-vascular therapy.

## Supplementary Material

Supplementary figures and table.

## Figures and Tables

**Table 1:** Baseline characteristics of the patients.

Characteristic	First-line immunotherapy No. (%)	First-line targeted therapy No. (%)	*p* value
Age			
≤60	40(48)	29(57)	0.298
>60	44(52)	22(43)	
Sex			
Male	62(74)	44(86)	0.087
Female	22(26)	7(14)	
Time from diagnosis to treatment			
≤1 year	60(71)	32(62)	0.294
>1 year	24(29)	19(38)	
Hemoglobin count			
<normal value	24(29)	19(38)	0.857
≥normal value	60(71)	32(64)	
Calcium			
≤normal value	73(87)	47(92)	0.262
>normal value	11(13)	4(8)	
KPS			
<80	2(2)	3(5)	0.528
≥80	82(98)	48(95)	
Platelet count			
≤normal value	69(82)	47(92)	0.054
>normal value	15(18)	4(8)	
Neutrophil count			
≤normal value	81(96)	48(94)	0.598
>normal value	3(4)	3(6)	
IMDC			
Low risk	22(26)	13(25)	0.940
Medium risk	55(65)	32(64)	
High risk	7(9)	6(11)	
cT stage			
T1	25(29)	14(28)	0.969
T2	26(30)	15(29)	
T3	15(18)	11(21)	
T4	5(6)	4(8)	
Tx	13(17)	7(14)	

**Table 2:** Single factor logistics regression analysis.

Characteristic	*p* value	OR	95%CI
Age (≤60 VS >60)	0.536	0.846	0.497-1.438
Sex (Male VS Female)	0.925	1.031	0.546-1.945
Time from diagnosis to treatment (≤one year VS >one year)	0.279	0.736	0.422-1.282
Hemoglobin count (<normal value VS ≥normal value)	0.569	1.171	0.679-2.020
Calcium (≤normal value VS >normal value)	0.399	0.692	0.294-1.629
KPS (<80 VS ≥80)	0.721	1.17	0.495-2.764
Platelet count (≤normal value VS >normal value)	0.053	0.384	0.159-0.925
Neutrophil count (≤normal value VS >normal value)	0.768	1.15	0.454-2.915
cT stage			
T1	0.655	0.887	0.523-1.502
T2	0.488	0.845	0.525-1.360
T3	0.211	0.657	0.340-1.268
T4	0.094	2.042	0.886-4.706
First-line Immunotherapy VS Second-line immunotherapy	0.002	0.411	0.235-0.721

**Table 3:** Targeted combination immunotherapy adverse reactions at different stages of treatment.

Treatment-related AEs	First-line	Second-line	*p* value	
Bone marrow suppression				
Grade 1-2	45(53)	32(63)	0.38	
Grade 3	21(25)	10(20)	0.60	
Grade 4	18(22)	9(17)	0.75	
Elevated liver enzymes				
Grade 1-2	30(36)	19(37)	1.00	
Grade 3	4(5)	6(12)	0.24	
Hypertension				
Grade 1-2	9(11)	7(14)	0.80	
Grade 3	3(4)	5(10)	2665	
Proteinuria	17(20)	10(20)	1	
Deep venous thrombosis	6(7)	4(8)	1	
Feeble	7(8)	5(10)	1	
Hand-foot syndrome	11(13)	8(16)	0.86	
