# Supplementary material for: Administering immunotherapy after anti-vascular targeted therapy improves overall survival of patients with metastatic clear cell renal cell carcinoma
Source: J Cancer. 2024 Jun 17;15(14):4527–33. doi: 10.7150/jca.96514 (PMC11242332; doi:10.7150/jca.96514)
Supplement: Supplementary file 1 — Supplementary figures and table. [file jcav15p4527s1.pdf]

Supplementary Figure1: The progression-free survival (PFS) was compared between first-line targeted therapy combined immunotherapy and first-line immunotherapy alone. (TI:TKI combined Immunotherapy, IO: Immunotherapy).

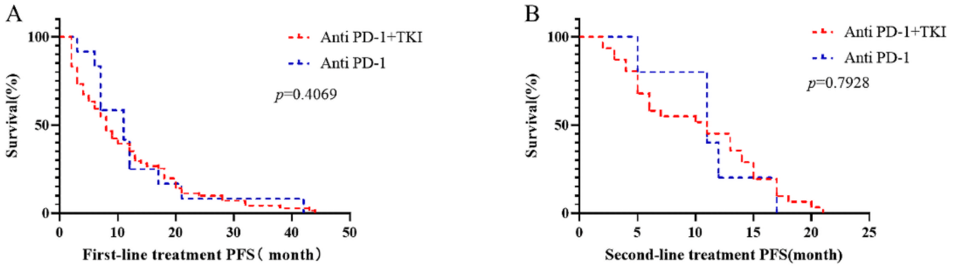

Supplementary Figure2: A: PFS was compared between first-line immunotherapy and first-line targeted therapy; B: The PFS of targeted combined immunotherapy after progression on first-line immunotherapy and targeted combined immunotherapy after progression on first-line targeted therapy were compared. (IO: Immunotherapy).

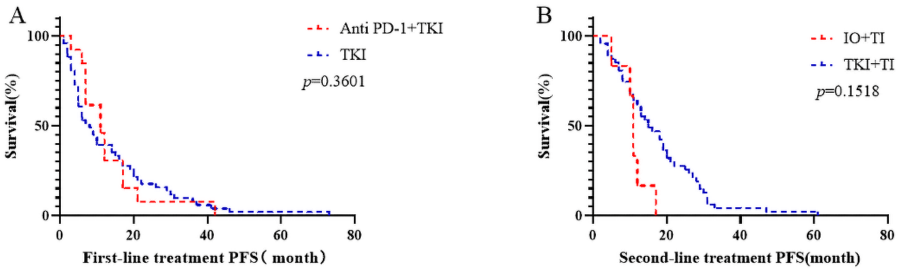

Supplementary table 1. Adverse reactions

Treatment-related AEs	First-line Immunotherapy group NO(%)		Second-line Immunotherapy group NO(%)		*p* value
	first-line	second-line	first-line	second-line	
Bone marrow suppression					
Grade 1-2	45(53)	50 (59)	25(49)	32(63)	0.93
Grade 3	21(25)	19(23)	12(24)	10(20)	0.97
Grade 4	18(22)	15(18)	14(27)	9(17)	0.96
Elevated liver enzymes					
Grade 1-2	30(36)	27(32)	16(31)	19(37)	0.88
Grade 3	4(5)	6(7)	3(6)	6(12)	0.82
Hypertension					
Grade 1-2	9(11)	11(13)	6(12)	7(14)	0.99
Grade 3	3(4)	4(5)	5(10)	5(10)	0.31
Proteinuria	17(20)	15(18)	12(24)	10(20)	0.96
Deep venous thrombosis	6(7)	5(6)	4(8)	4(8)	0.97
Feeble	7(8)	8(10)	5(10)	5(10)	0.99
Hand-foot syndrome	11(13)	9(11)	6(12)	8(16)	0.85
